# Important miRs of Pathways in Different Tumor Types

**DOI:** 10.1371/journal.pcbi.1002883

**Published:** 2013-01-24

**Authors:** Stefan Wuchty, Dolores Arjona, Peter O. Bauer

**Affiliations:** 1 National Center of Biotechnology Information, National Library of Medicine, National Institutes of Health, Bethesda, Maryland, United States of America; 2 GeneDx Inc., Gaithersburg, Maryland, United States of America; 3 Neuro-Oncology Branch, National Cancer Institute, National Institutes of Neurological Disorder and Stroke, National Institutes of Health, Bethesda, Maryland, United States of America; Princeton University, United States of America

## Abstract

We computationally determined miRs that are significantly connected to molecular pathways by utilizing gene expression profiles in different cancer types such as glioblastomas, ovarian and breast cancers. Specifically, we assumed that the knowledge of physical interactions between miRs and genes indicated subsets of important miRs (IM) that significantly contributed to the regression of pathway-specific enrichment scores. Despite the different nature of the considered cancer types, we found strongly overlapping sets of IMs. Furthermore, IMs that were important for many pathways were enriched with literature-curated cancer and differentially expressed miRs. Such sets of IMs also coincided well with clusters of miRs that were experimentally indicated in numerous other cancer types. In particular, we focused on an overlapping set of 99 overall important miRs (OIM) that were found in glioblastomas, ovarian and breast cancers simultaneously. Notably, we observed that interactions between OIMs and leading edge genes of differentially expressed pathways were characterized by considerable changes in their expression correlations. Such gains/losses of miR and gene expression correlation indicated miR/gene pairs that may play a causal role in the underlying cancers.

## Author Summary

We assume that a network of physical interactions between miRs and genes allows us to determine miRs that influence the expression of whole pathways in different tumor types. Specifically, we represented each pathway by an enrichment score and an array of miRs counting the number of genes in the pathway a given miR can bind. Despite the different nature of the considered tumor types, we obtained a large set of overlapping miRs using a machine-learning algorithm. Such associated miRs were enriched with literature-curated cancer and differentially expressed miRs and also coincided well with clusters of miRs that were experimentally indicated in numerous other cancer types. Focusing on such sets of miRs we observed that interactions with genes in differentially expressed pathways were characterized by massive gains/losses of expression correlations. Such drastic changes of miR and gene expression correlation indicate miR/gene pairs that may play a causal role in the underlying cancers.

## Introduction

MicroRNAs (miRs) are small non-coding RNAs that interact with their gene target coding mRNAs. Such small RNAs putatively inhibit translation by direct and imperfect binding to the 3′- and 5′-untranslated regions (UTR) [1] and exert expression control with other regulatory elements such as transcription factors [2], [3], [4].

The elementary role of miRs in gene expression has been indicated in tissue- and organ-specific development [5]. miRs also play an important role in tumors [6], [7], [8], where over-expressed miRs might diminish the level of expression of targeted tumor suppressor genes [9]. In turn, miRs may act as tumor suppressors, when their down-regulation leads to enhanced expression of targeted oncogenes [10] or are involved in various steps of the metastatic process [11]. Generally, aberrant expression of miRs in cancers can arise from the deletion or mutation as well as methylation of miR coding regions [12]. Furthermore, miRs may be located in common breakpoint regions and genomic areas of amplification and loss of heterozygosity [13]. Such alterations of miR-expression levels have been implicated in the de-regulation of critical players in major cellular pathways, modifying the differentiation, proliferation and survival of tumor cells. For example, miR-7 and miR-221/222 have been shown to be involved in the activation of the Akt and epidermal growth factor receptor (EGFR) signaling pathways in gliomas [14], [15] while miR-34a was found to be a key regulator of p53 [16].

To provide a better understanding of the involvement of miRs in pathways, we computationally determined miRs that are significantly associated with molecular pathways. In particular, we utilized gene expression profiles to determine a pathway specific enrichment score in diverse cancer types, such as glioblastomas, ovarian and breast cancers. Using data of physical interactions between miRs and the 3′UTR of mRNAs we counted the numbers of leading edge genes (LEG) in each pathway that were targeted by a given miR. We assumed that the topology of interactions between LEGs of pathways and miRs allows an assessment of the tumor-specific importance of the given miR for the expression of the underlying pathways. Therefore, we used a machine learning approach to fit pathway-specific enrichment scores as a function of the corresponding number of LEGs that were targeted by an array of miRs. Despite the diversity of the underlying cancer types, we obtained a large, overlapping set of important miRs (IM) that significantly influenced the regression process in all cancer types considered. Furthermore, IMs that were important for an increasing number of pathways were enriched with literature curated cancer miRs and differentially expressed miRs. Such sets of IMs also coincided well with clusters of miRs that were experimentally indicated in numerous other cancer types. Focusing on such an overlapping set of overall important miRs (OIM) in glioblastomas, ovarian and breast cancers, we investigated their interactions to LEGs in differentially expressed pathways. We observed that such interactions were characterized by considerable changes in their expression correlations. Such gains or losses of expression correlations indicated OIM/LEG pairs that may influence expression changes in the underlying pathways.

## Methods

### Matching gene and miR expression data

Using The Cancer Genome Atlas (TCGA, http://cancergenome.nih.gov/), we utilized 77 glioblastoma samples and 10 non-tumor control samples that provided matching gene and miR expression profiles. We also used 77 samples of ovarian cancer and 8 non-cancer tumor samples, as well as 79 breast cancer and 19 non-cancer control samples.

Comparing disease and control samples, we determined differentially expressed miRs by a Student's t-test if FDR<0.01. Accordingly, we found 164 differentially expressed miRs in GBMs, 282 in ovarian and 82 in breast cancers.

### Literature curated cancer-miRs

We collected overlapping sets of 35 oncomiRs, 42 tumor suppressor- miRs [6], [9], [17], [18], [19], [20], [21] and 32 miRs that were involved in metastasis [11], [19], [20], [21], [22] (Fig. S1). The HMDD database [23] collects reports from the literature that experimentally indicated a miRs involvement in different tumor types. Specifically, we utilized sets of 45 miRs in glioblastomas, 81 in ovarian and 125 in breast cancer.

### Pathway information

As a source of reliable protein pathway information, we used 429 annotated pathways from the Reactome database [24].

### miR-mRNA interactions

Utilizing human specific data from PicTar [25], miRanda [26], [27] and TargetScanS [28] we assembled 48,939 interactions between 386 miRNAs and 6,725 mRNAs, demanding that each interaction was reported by at least two sources [29]. All interaction pairs are presented in Table S1.

### Determination of important miRs (IM)

Using gene expression data of a cancer type, we applied GSEA [30] to calculate a normalized enrichment score of each pathway. We represented each pathway by a profile of miRs that reflected the number of leading edge genes (LEG) in the underlying pathway a given miR interacts with. Focusing on a given miR we normalized such numbers by a Z-score averaging over all pathways. Finally, we used random forest algorithm [31] to perform a regression of the pathways normalized enrichment scores as a function of the miR profiles of Z-scores. In each of 10,000 regression trees, we randomly sampled 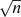 of all *n* miRs and 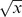 of all *x* pathways [21], [29]. As for the assessment of a miR's importance for each pathway in the fitting process, we permuted enrichment scores and the number of targeted LEGs, calculating randomized local importance values for each miR/pathway pair. We repeated the randomization process 100 times and constructed null-distributions of randomized importance scores for each miR/pathway pair. Fitting such distributions with a Z-test, we calculated P-values for each miR/pathway pair. We corrected for multiple testing by calculating the corresponding false discovery rate (FDR) [32] and defined an important miR (IM) of a pathway if FDR<0.01.

### Enrichment analysis

We grouped important miRs (IM) according to their number of pathways. Specifically, we represented each group by 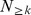 IMs that had at least *k* pathways. In each group we calculated the number of IMs with a certain feature *i* (*i.e.* being differentially expressed or a cancer miR), 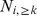. Randomly assigning feature *i* to IMs we defined 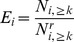 as the enrichment of IMs with feature *i* where 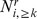 was the corresponding random number of IMs with feature *i* among all 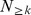 IMs. After averaging *E_i_* over 10,000 randomizations *E_i_>1* pointed to an enrichment and *vice versa*, while *E_i_∼1* indicated a random process [33]. Analogously, we determined the enrichment of differentially expressed pathways as a function of the number of their IMs.

### Change of expression correlations

Assuming *N_D_* cancer and *N_C_* non-tumor control samples, we calculated Pearson's correlation coefficient of an interacting miR *i* and gene *j* in the disease (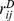) and control (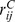) samples. Subsequently, we Fisher transformed correlation coefficients into a Z-score reflecting the difference of correlation coefficients defined as 
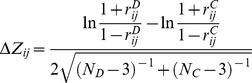
. Therefore, a positive ΔZ corresponded to a gain of correlation in the disease case and *vice versa*.

## Results

Utilizing The Cancer Genome Atlas (TCGA, http://cancergenome.nih.gov/), we searched for samples in cancer types that provided matching gene and miR expression profiles of each sample. While we obtained 77 GBM and 10 non-cancer brain control samples, we identified 77 samples of ovarian cancer and 8 non-ovarian cancer samples, as well as 79 breast cancer and 19 non-breast cancer control samples. As a reliable source of canonical protein pathway information we used 429 pathways from the Reactome database [24].

### Determination of pairs of important miRs (IM) and pathways

**Figure 1:**
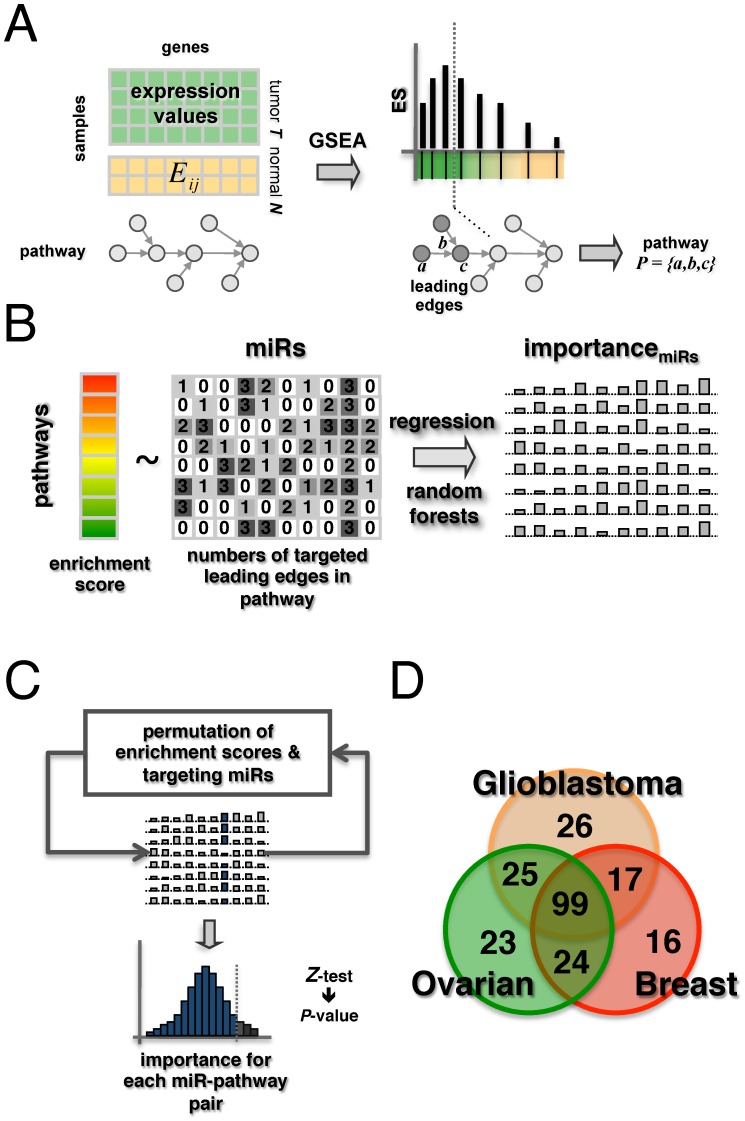
Prediction of important miRs. In (**A**) we utilized gene expression profiles of cancer and non-cancer control samples and determined normalized GSEA enrichment scores of molecular pathways. In addition, we represented each pathway by the corresponding set of leading edge genes (LEG). (**B**) We fitted pathway-specific enrichment scores as a function of the corresponding number of LEGs that are targeted by a given miR. Using the random forest algorithm we obtained importance scores of each miR. (**C**) To assess the statistical significance of a miR's importance we performed permutation tests, randomizing both enrichment scores and the number of targeted LEGs. Building random distributions of importance scores, we utilized a Z-test to determine corrected P-values of each miR/pathway pair. (**D**) While we found 167 important miRs (IM) in GBMs, 171 in ovarian cancer and 156 in breast cancer (FDR<0.01), we observed large overlaps between these sets of IMs.

In the first step of our procedure (Fig. 1A), we applied Gene Set Enrichment Analysis (GSEA) [30] to determine a normalized enrichment score of each pathway, comparing expression profiles in cancer cases to their non-cancer controls. Accounting for the expression characteristics of different cancer types, we represented each pathway by ‘leading edge genes’ (LEG), a subset of genes that significantly drove the enrichment of a given pathway in the disease cases [30]. Furthermore, we assembled 48,939 interactions between 386 miRs and 6,725 mRNAs (Table S1). Pooling such miR-gene interaction data from PicTar [25], miRanda [26], [27] and TargetScan [28], we demanded that each interaction was reported by at least two sources [29]. Considering each pathway as a set of LEGs, we counted the number of such genes that a given miR interacted with. Consequently, each pathway was further represented by a miR interaction profile, indicating the number of LEGs in a pathway a given miR interacted with (Fig. 1B). Averaging over all pathways, we normalized miR-specific entries in this matrix by a Z-score. Representing each pathway by its normalized enrichment score, we applied the random forest algorithm, allowing the calculation of an importance value for each miR/pathway pair. Such an importance measure reflects the impact of the given miR on the fitting process of the underlying pathway's enrichment score. To assess the statistical significance of local importance scores we resorted to permutation tests (Fig. 1C). Randomizing both pathway enrichment scores and the miRs numbers of targeted LEGs we generated null-distributions of importance scores for each miR/pathway pair. Utilizing a Z-test we determined P-values and observed a pair of an important miR (IM) and a pathway if FDR<0.01 [32].

In glioblastomas, we found a total of 2,320 significant pairs between 167 IMs (49.6% out of all miRs that interacted with LEGs in 429 pathways) and 265 pathways (61.8% out of all 429 pathways). Furthermore, we observed that the set of pathways was significantly enriched with differentially expressed pathways as provided by GSEA (FDR<0.01) applying Fisher's exact test (P<10^−12^). Similarly, we found 2,564 pairs between 171 IMs (50.3%) and 322 pathways (75.1%) in ovarian cancer (P<10^−7^) while 156 IMs (47.3%) were linked to 309 pathways (72.0%) through 2,041 pairs in breast cancer (P<10^−9^). For a complete list of all IM/pathway pairs see Tables S2, S3, S4. In Fig. 1D, we observed that sets of IMs largely overlapped, allowing us to find 99 overall important miRs (OIM), corresponding to 59.2% of IMs in GBM, 57.9% in ovarian and 63.5% in breast cancer. In turn, we also found that pathways overlapped strongly (Fig. S2A) with 182 pathways present in all cancer types considered, a value that translated into 68.7% of pathways in GBMs, 56.5% in ovarian and 58.9% in breast cancers. Furthermore, we observed a small overlap of 98 IM-pathway pairs that appeared in all cancer types considered (Fig. S2B, Table S5).

### Statistics of important miRs and pathways

Since we determined the impact of each interacting miR on the fit of each pathway's enrichment score, an IM may be important to more than one pathway and *vice versa*. In Fig. S3A, we observed a logarithmic decay in the frequency distribution of the number of pathways an IM targeted in all cancer types. In turn, the frequency distribution of the number of IMs a given pathway is significantly linked to decreased exponentially as well (inset, Fig. S3B).

Obtaining auxiliary cancer-related information, we collected 72 cancer-related miRs from the literature, consisting of overlapping sets of 35 onco-, 42 tumor suppressor- and 32 metastamiRs [6], [9], [17], [18], [19], [20], [21] (Fig. S1). Furthermore, we utilized the HMDD database [23] pooling experimental evidence that a miR was involved in given cancer types. We also determined differentially expressed miRs with a t-test (FDR<0.01) [32] using miR expression profiles of glioblastomas, ovarian and breast cancer. In Table S6 we ordered IMs according to their corresponding number of pathways in each cancer type. Specifically, IMs that were linked to an increasing number of pathways seemed to be enriched with literature curated cancer miRs, tend to be differentially expressed and experimentally indicated in the given cancer types.

On a more quantitative basis, we grouped IMs according to their number of pathways in a given cancer type. In groups of IMs that were linked to at least *k* pathways we determined the number of literature-curated miRs. In a null-model, we randomly picked sets of literature-curated miRs and determined their enrichment in each group as the ratio of the observed and expected numbers. Fig. S4A suggests that groups of IMs with increasing numbers of pathways tend to be enriched with cancer miRs in all given cancer types. Analogously, we determined the enrichment of differentially expressed miRs and observed that such groups of IMs were predominantly enriched with differentially expressed miRs as well (inset, Fig. S4A). Similarly, we calculated the enrichment of differentially expressed pathways as a function of the number of IMs of a given pathway. Distributions in Fig. S4B suggested that pathways with an increasing number of IMs had a heightened tendency to be differentially expressed in all tumor types considered.

### Comparison of IMs to miRs implicated in other cancer types

**Figure 2:**
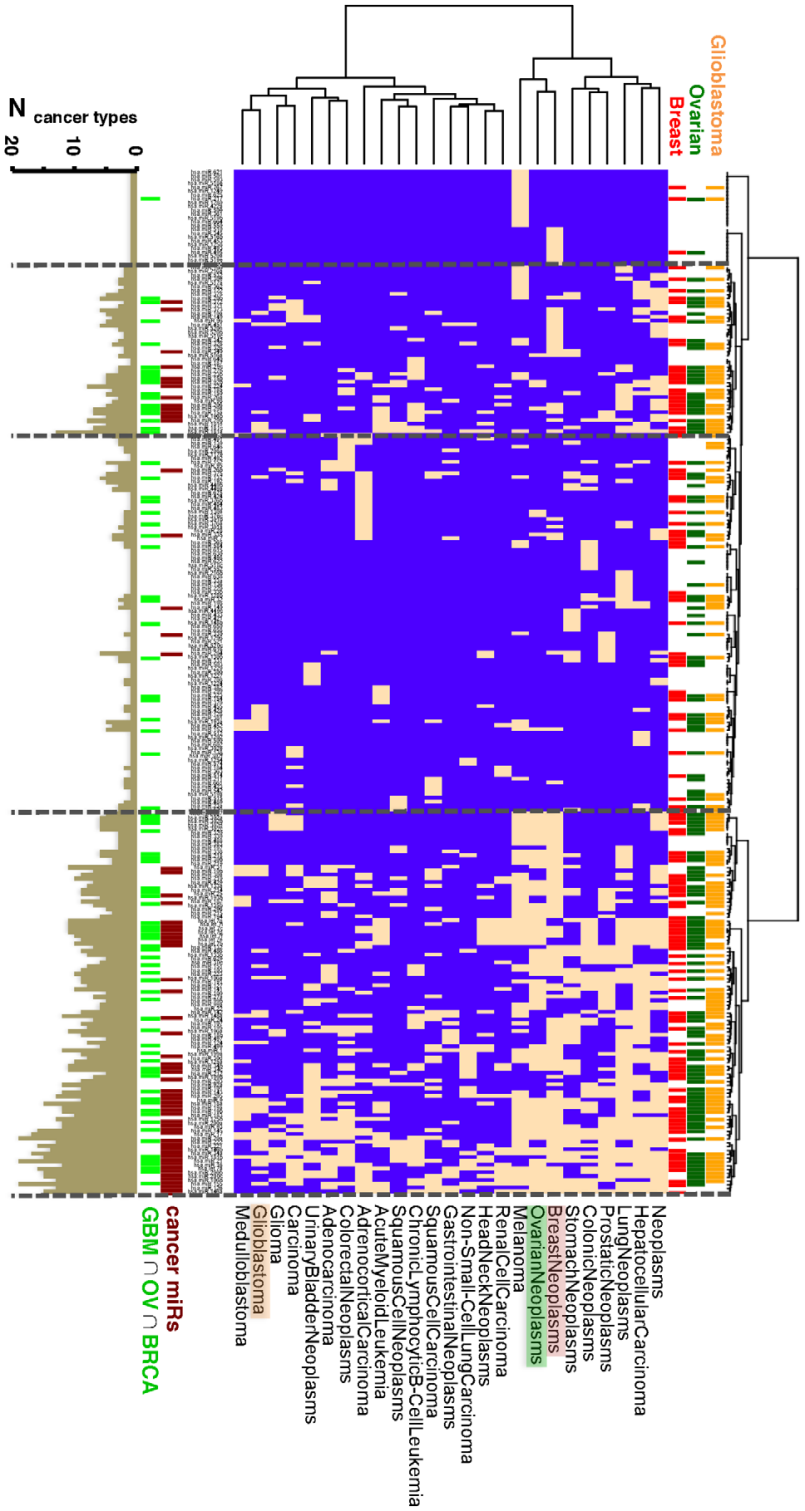
Comparison to experimentally determined miR involvement in various cancer types. In the heatmap of 25 different cancer types we showed miRs that were experimentally indicated in the underlying tumor types (peach boxes). We observed two large clusters (dashed lines), consisting of most frequently indicated miRs (histogram). Important miRs (IM) found in glioblastomas, ovarian and breast cancers separately corresponded well to such clusters. Such a trend was reinforced by the overlaps of these sets and matched the placement of literature curated cancer miRs as well.

Utilizing data from the HMDD database [23] we collected information about miRs that were experimentally found to play a role in more than 90 cancer types. Focusing on 25 cancer types with at least 25 different, implicated miRs (including glioblastoma, ovarian and breast cancer) we constructed a bipartite matrix, indicating if a given miR was experimentally reported in a certain cancer type. Ward-clustering such a binary matrix, we observed two large clusters of miRs (Fig. 2). Counting the number of different cancer types a miR was experimentally found in, we observed that such clusters consisted of the most frequently indicated miRs (histogram, Fig. 2). Therefore, we expected that such clusters may be enriched with IMs. Indeed, our separate sets of IMs in glioblastoma, ovarian and breast cancer overlapped well with this general pattern of miR involvement in different tumor types. Applying a hypergeometric test we further checked if IMs were enriched among miRs that appeared in at least 3 different cancer types. Indeed, 106 IMs in GBMs occurred in such a set of miRs (P<10^−5^), while we found 107 in ovarian (P<10^−4^) and 100 in breast cancers (P<10^−4^). Focusing on our set of 99 overlapping, overall important miRs (OIM) in GBMs, ovarian and breast cancers we also observed a significant overlap of 74 miRs (P<10^−5^). Furthermore, literature curated cancer miRs were largely placed in previously mentioned clusters as well. In particular, 38 cancer miRs overlapped with our set of 99 OIMs (P<10^−10^), suggesting that OIMs may play a central role in different cancer types.

### Analysis of overall important miRs (OIM)

**Figure 3:**
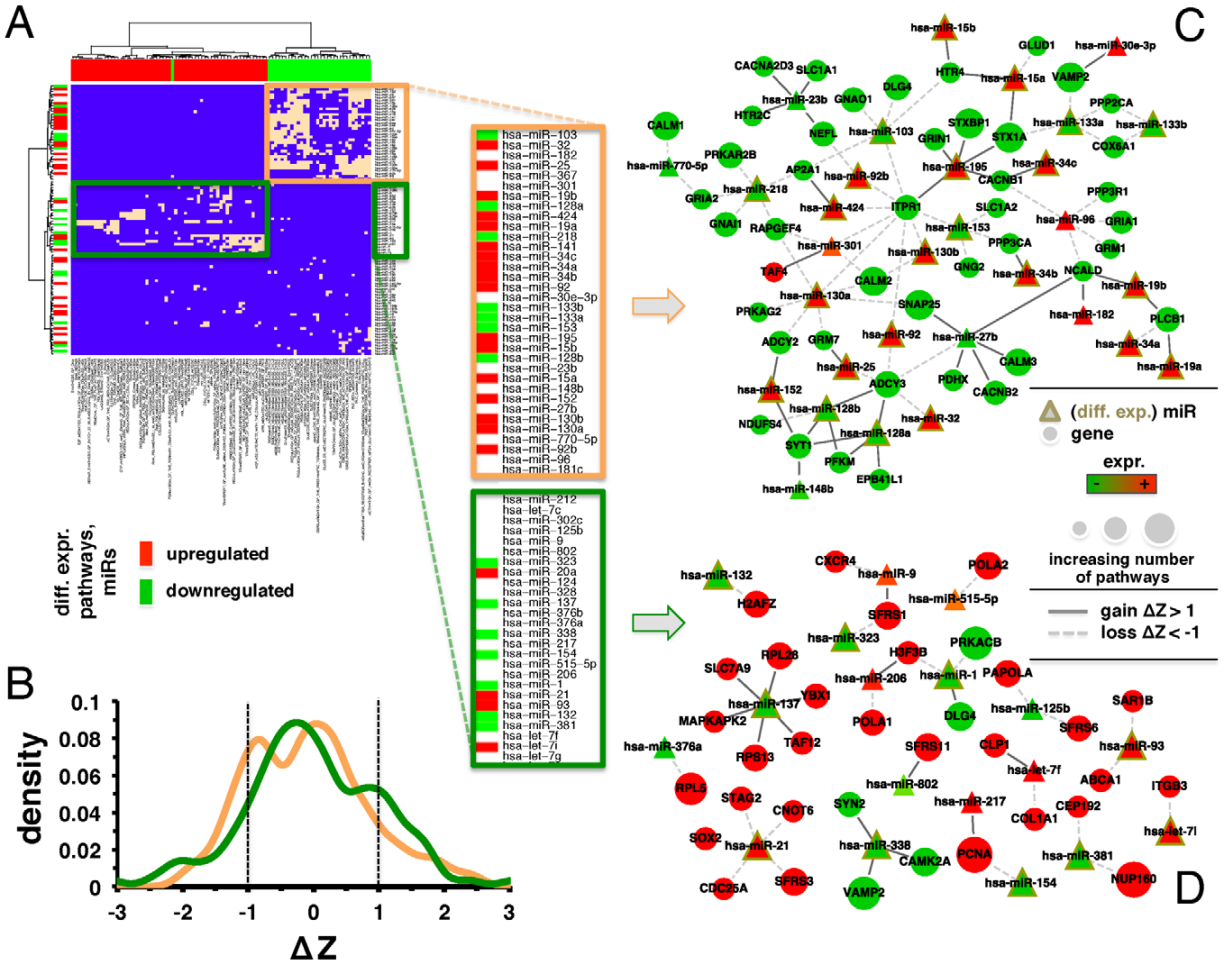
Analysis of correlation change in glioblastomas. (**A**) Focusing on a set of 99 overlapping overall important miRs (OIM) in GBMs, ovarian and breast cancers, we indicated OIMs and their corresponding differentially expressed pathways in GBMs (peach boxes). In such a binary matrix we observed two large clusters that distinguished largely between either up- or down regulated pathways (orange, green boxes). In (**B**) we calculated the change of expression correlation, ΔZ, for all pairs of OIMs in such clusters and their interacting leading edge genes (LEG) in the corresponding pathways. In particular, we observed multimodal distributions with local peaks around ΔZ = ±1.0 (dashed lines). In (**C**) and (**D**) we mapped all interactions between OIMs and LEGs of pathways that corresponded to the observed clusters. Specifically, we only accounted for interactions with a correlation change |ΔZ|>1.0.

Utilizing such an overlapping set of 99 OIMs, we focused on connections to differentially expressed pathways and found a total of 93 pathways in glioblastoma, 55 in ovarian and 87 in breast cancers. Mapping the corresponding links between OIMs and these pathways in glioblastoma we constructed a binary matrix. Ward clustering allowed us to obtain two large clusters of either up- or down-regulated pathways that strongly corresponded to two groups of largely down- or up-regulated, differentially expressed OIMs (Fig. 3A). Down-regulated pathways mostly revolved around neurotransmitter specific pathways while up-regulated pathways covered prominent signaling, regulation and transcription functions (see for an enlargement). As for ovarian (Fig. S6A) and breast cancers (Fig. S7A), we obtained similar results. Notably, we only observed interactions between OIMs and up-regulated pathways in ovarian cancers that largely revolved around signaling and regulation functions.

Using such pairs of OIMs and pathways in GBMs, we retrieved all interactions between OIMs and LEGs in the corresponding pathways that were placed in the previously found clusters. Merging gene and miR expression data, we calculated Pearson's correlation coefficients using gene and miR expression profiles in glioblastoma and non-tumor control samples. As a measure of the difference between expression correlation coefficients in the disease (*r^D^*) and non-tumor control cases (*r^C^*) we Fisher-transformed correlation coefficients into Z-scores and calculated the corresponding change in correlation, ΔZ. A negative/positive value of ΔZ indicates a loss/gain of correlation in the disease case. Focusing on interactions between OIMs and the corresponding LEGs of pathways in these clusters we observed bimodal distributions of ΔZs in glioblastoma (Fig. 3B). Notably, interactions between OIMs and LEGs that corresponded to down-regulated pathways and predominantly up-regulated miRs were characterized by a peak at ΔZ = −1.0, pointing to a loss of expression correlation. Focusing on miR/gene interactions in the cluster of up-regulated pathways and largely down-regulated miRs we observed a peak at ΔZ = +1.0, pointing to a gain of correlation. Analogously, we obtained such distributions for pairs of OIMs and LEGs in ovarian (Fig. S6B) and breast cancer (Fig. S7B).

Focusing on GBMs, we mapped all interactions between OIMs and LEGs we found in the corresponding clusters if their correlation change was |ΔZ|>1.0. As for the cluster that revolved around down-regulated pathways and up-regulated OIMs (Fig. 3C), we observed many interactions between differentially expressed OIMs and ITPR1 (inositol 1,4,5-trisphosphate receptor type 1) with losses of expression correlations. Overall important miRs mapped in this analysis included miR-34a, -27b, -128ab and -15b. Focusing on the cluster composed by down-regulated pathways and largely up-regulated OIMs (Fig. 3D), we found miR-21 and let-7i in interactions with losses of expression correlation and miR-137 in interactions that gained expression correlation.

We mapped miRs and associated pathways in ovarian (Fig. S6C) and breast cancers as well (Fig. S7CD). While we found a strong presence of signaling, transcription and translation related pathways in ovarian cancers, we also observed pathways that revolved around transcription factor E2F and the SFRS1 protein.

Focusing on a cluster of up-regulated pathways in breast cancers and largely up-regulated miRs (Fig. S7C) we found down-regulated AKT3 that was interacting with a couple of up-regulated miRs. These results are discussed below (see Discussion).

## Discussion

Although a growing appreciation of the importance of miRs in cancers is emerging, much remains unknown about their regulatory impact. Current knowledge appears rather scattered, focusing on single interactions between miRs and target genes of interest in a given cancer type. Here, we chose a different approach by utilizing pairwise interactions between miRs and target genes to identify combinations of important miRs (IM) and pathways in a given cancer type.

A major criterion that may influence our results is the accuracy of computational methods that predict interactions between miRs and the UTRs of genes. Since such computational approaches suffer from false positives, we chose results of three different algorithms and demanded that each interaction was at least predicted twice, potentially allowing us to limit spurious signals [34].

We modeled the expression change of pathways comparing sets of cancer to non-tumor control cases as a function of the number of interactions between leading edge genes that drive the expression of a given pathway and miRs. We stress our initial assumption that the mere number of targeted LEGs in a pathway is a reasonable proxy to model the expression change of pathways in a disease, therefore allowing us to capture tumor specific effects. Although our approach did not account for any expression levels of miRs in given tumor types, we assume that the expression change of pathways is not only a matter of leading edge genes but the binding miRs as well. As such, we modeled expression change as a skeleton of miR interactions. Since such links strongly influence the flow of molecular information, we conclude that the consideration of miRs expression putatively won't override results that were largely imposed by the underlying topology of miR interactions.

Furthermore, such an approach allows us to determine combinations of important miRs that potentially influence such expression changes through their targeted LEGs in the given pathways. Utilizing data of diverse cancer types, such as glioblastomas, ovarian and breast cancers, we clearly observed largely overlapping sets of IMs that were predominantly linked to differentially expressed pathways. Confirming our initial hypotheses, IMs with many pathways were predominately enriched with literature-curated cancer miRs and differentially expressed miRs. Besides, such pathway specific connections may be harnessed to predict meaningful sets of miRs that play a role in the underlying cancers. Notably, overall important miRs (OIM) in all cancer types coincided well with the most frequently indicated cancer –related miRs in different cancer types, indicating the relevance of our predictions. While the consideration of miR expression levels may change the number of IMs, such observations strongly suggest that a diminished set of OIMs will continue to show similar characteristics.

Focusing on specific details of glioblastomas, ovarian and breast cancers, such cancer types are typically stratified by certain subtypes as indicated by subtle changes in gene expression profiles. While we acknowledge that pairs of pathways and important miRs may vary, we don't expect that the sets of IMs will dramatically change: considering that completely different cancer types with significant differences in their gene expression profiles provided largely overlapping sets of IMs, we expect that results that account for subtype information will be largely robust.

Focusing on our set of 99 OIMs, we identified all interactions to LEGs in differentially expressed pathways. Comparing non-tumor control to disease cases, such interactions suffered partially from a massive loss of (anti-) correlation that were indicated by multimodal distributions of expression correlation changes. Dramatic changes of the expression correlation of interactions may therefore be considered to significantly influence the expression of LEGs, contributing to the perturbation of pathways in the underlying cancer types.

As for qualitative observations of such OIM-LEG pairs we found that many differentially expressed miRs appeared interacting with ITPR1 in GBMs (Fig. 3C). This receptor1 is central to many signaling GBM-relevant pathways, including NGF and Plc-**γ**1 signaling pathways as well as insulin regulation and diabetes related pathways. miR-34a has been found to play an important role in glioblastoma as a tumor suppressor [16], [35] while being a mediator of p53 [14], [36], [37], [38], [39] in an interaction with a loss of expression correlation. Important targets of miR-34a included members of the Notch family and the oncogene c-met [40]. Specifically, we found an association of miR-34a with phospholipase C (PLCB1), which has recently been identified as a regulator of glioma cell migration [41].

The result of miR-27b was rather unexpected, since this miR has been reported up-regulated in gliomas [42]. However, the observed discrepancy may result from the experimental setup where the up-regulated miR-27b might have resulted from an inflammatory reaction [43] and originated from other than the glioma cells. Moreover, miR-27b has been identified as a pro-angiogenic miR in endothelial cells [44] and found to be involved in tumor angiogenesis [45]. Regarding the up-regulation of miR-27b in glioma cells, cell culture conditions used in [42] promote cell differentiation (medium containing fetal bovine serum) that may artificially affect the miRs expression profile. Therefore, we believe that the down-regulation of miR27b and its effects on calcium metabolism (CALM3, CACNB2) and exocytosis-related (SNAP25) genes reflect the actual situation in GBMs.

The down-regulation of miR-128ab in human glioma and glioblastoma cell lines has previously been reported [46] to increase the expression of ARP5, Bmi-1 and E2F-3a, promoting neural stem cells renewal and regulate cell-cycle progression [46]. Beside miR-128ab being important regulators of brain cell proliferation, we indicated that miR128ab may also affect expression of genes involved in energy metabolism (PFKM) and transmembrane signal transduction (SYT1, EPB41, ADCY3).

miR-15b has been identified as an inhibitor of glioma growth while cyclin E1 has been found as a target of miR-15b, suggesting its role in cell cycle regulation [47]. Here, we observed that serotonin receptor 4 (HTR4) was down-regulated in glioblastoma samples, a process that is associated with up-regulation of miR-15b.

The cluster composed of down-regulated pathways and largely up-regulated OIMs (Fig. 3D) revealed miR-21, let-7i, and miR-137 to be involved in interactions with losses and gains of expression correlation, respectively. Putatively, miR-21 works as an ‘oncomiR’, decreasing apoptosis in malignant cells while down-regulated miR-137 is involved in the differentiation of glioma stem cells [48]. Implicated in the development of glioblastomas [49], [50], knockdown of miR-21 leads to reduced cell proliferation, invasiveness, tumorigenicity and increased apoptosis [49], [50], [51]. Furthermore, miR-21 was reported to be involved in at least three tumor-suppressive pathways including mitochondrial apoptosis, p53 and TGF-β [50], [52], [53], [54] pathways. Our results revealed further cancer-relevant target genes including STAG2, CNOT6, SOX2, CDC25A and SFRS3 (Fig. 3D). Specifically, STAG2 encodes a subunit of cohesion, a multimeric protein complex required for cohesion of sister chromatids after DNA replication. Furthermore, STAG2 is cleaved at the metaphase-to-anaphase transition to enable chromosome segregation [55], [56], [57]. Chromosomal instability, which leads to aneuploidy, loss of heterozygosity, translocations and other chromosomal aberrations is one of the hallmarks of cancer [57]. Robust STAG2 expression has been shown in non-neoplastic tissues while significant fractions of glioblastomas had completely lost expression of STAG2 [58], suggesting that miR-21 may have both oncogenic and tumor-suppressive effects. A link between miR-21 and the p53 pathway could be CNOT6 (Ccr4a), a deadenylase subunit of the Ccr4-Not complex that is involved in mRNA degradation [59]. Ccr4a, together with Ccr4b, has been identified as a key regulator of insulin-like growth factor-binding protein 5, mediating cell cycle arrest and senescence through the p53-dependent pathway [60], [61]. Moreover, CNOT6 plays an important role in chemotherapy resistance to cisplatin through down-regulation of DNA-damage response by targeting Chk2 [62]. miR-21 expression was shown up-regulated in response to ionizing radiation while the inhibition of miR-21 enhanced the radiation-induced glioblastoma cell growth arrest and increased the level of apoptosis. While this effect may be mediated by CDC25A [63], our results suggested that CDC25A was targeted by miR-21 (Fig. 3D). Additionally, Cdc25A appears to be a promising therapeutic target in glioblastomas as its levels were reported to correlate with Ki-67 labeling index [64]. Another target gene that we identified to be controlled by miR-21, SFRS3, is a pro-oncogene involved in mRNA and rRNA processing. Furthermore, SFRS3 has been reported as a critical factor for tumor induction, progression and maintenance [65], [66]. Lastly, the association of miR-21 with SOX2, a marker for undifferentiated and proliferating cells with up-regulated expression in glioblastomas [67] further underlined the importance of miR-21 for the pathogenesis of these tumors.

Let-7 appears to be a tumor suppressor while inhibiting K-ras and C-myc [68], [69]. In glioblastomas, overexpression of let-7 has been shown to decrease cell proliferation [70]. We found a link between let-7i and integrin β3 (ITGB3) whose pro-apoptotic role has been reported in glioma cells [71].

miR-137 is also a putative tumor suppressor and is down-regulated in gliomas through a DNA hypermethylation mechanism [48]. Cooperating with miR-124, miR-137 may suppress expression of phosphorylated Rb and CDK6 while inducing cell cycle arrest at G0/G1 in glioma cells [48]. Our results further suggested glioma relevant targets that are involved in AKT-mTOR signaling (MAPKAPK2 and YBX1) (Fig. 3D). The significance of other associated partners such as genes that encode ribosomal proteins RPL28 and RPS13 remains to be established.

Mapping OIMs and their pathways in ovarian cancer revealed interactions between several miRs and transcription factor E2F and particularly between E2F3 and miRs-148b, -124 and -34a (Fig. S6C). Indeed, miR-34a was shown to epigenetically govern the expression of E2F3 through methylation of its promoter [72]. In our analysis, miR-132 and miR-212 gain expression correlation in interactions with SFRS1, a proto-oncogene that is involved in pre-mRNA splicing with the ability to change the splicing patterns of crucial cell cycle regulators and suppressor genes. Of particular interest is the observation that SFRS1 is up-regulated in many cancer types and therefore a potential target for cancer therapy [73]. Importantly, the role of these miRs and their interactions with target genes in ovarian cancers is not well understood. However, indications exist that both miRs that share a seed sequence may play a role since both miRs were found to be down-regulated by promoter methylation that contributes to pancreatic cancers [74].

The down-regulation of AKT3 upon interaction with several up-regulated miRs was the highlight observation in the cluster of up-regulated pathways in breast cancers (Fig. S7D). AKT kinases are regulators of cell signaling in response to insulin and growth factors and are involved in a wide variety of biological processes including cell proliferation, differentiation, apoptosis, tumorigenesis as well as glycogen synthesis and glucose uptake. In our analysis, we found that AKT3 interacted with miRs-181ac, gaining expression correlation, while miR15a, -16 and -20a lost expression correlations with their target genes. In particular, miR-15a and -16 were already indicated as relevant in different cancers [75]. Furthermore, members of the miR-181 family were shown to induce sphere formation in breast cancer cells [76].

## Supporting Information

Figure S1 **Overlaps of sets of onco-, tumorsuppressor- and metastamiRs.** Venn diagram of the overlaps of 35 onco-, 42 tumorsuppressor- and 32 metastamiRs, totaling 72 cancer-related miRs.
(PDF)

Figure S2 **Overlaps of pathways and pairs of important miRs/pathways.** **(A)** While we found 365 pathways in GBMs, 322 miRs in ovarian cancer and 309 in breast cancer (FDR<0.01), we observed large overlaps between these sets. **(B)** Focusing on overlapping pairs of important miRs and pathways that appeared in all cancer types considered, we observed a small overlap.
(PDF)

Figure S3 **Statistics of important miRs and their pathways.** In **(A)** we counted the number of pathways of each important miRs (IM), allowing us to find a logarithmic decay in such a frequency distribution. **(B)** In turn, we determined the number of IMs of each pathway, indicating an exponential decay in the corresponding frequency distribution.
(PDF)

Figure S4 **Enrichment analyses.** In **(A)** we determined the enrichment of cancer miRs and differentially expressed miRs in groups of important miRs (IM) that have a certain number of pathways. Specifically, we observed that IMs with increasing number of pathways were enriched with literature curated cancer miRs as well as differentially expressed miRs (inset) in all cancer types considered. **(B)** In turn, we grouped pathways in sets that have at least a certain number of IMs. Determining the enrichment of differentially expressed pathways in such groups, we found that pathways with an increasing number of IMs tend to be differentially expressed.
(PDF)

Figure S5 **Enlargement of** **Fig. 3A** **in the main paper.**
(PDF)

Figure S6 **Analysis of correlation change in ovarian cancer.** **(A)** Focusing on our set of 99 overall important miRs (OIM), we indicated if such miRs were linked to differentially expressed pathways in ovarian cancer (peach boxes). We observed a large cluster that corresponded to down regulated pathways (orange box). In **(B)** we calculated the change of expression correlation, ΔZ, for all pairs of OIMs in this cluster and the interacting leading edge genes in the corresponding pathways, indicating local peaks around ΔZ = ±1.0 (dashed lines). In **(C)** we mapped all such interactions between OIMs and leading edge genes if they had a correlation change |ΔZ|>1.0.
(PDF)

Figure S7 **Analysis of correlation change in breast cancer.** **(A)** Focusing on our set of 99 OIMs, we indicated if such miRs were linked to differentially expressed pathways in breast cancer (peach boxes). Specifically, we observed two large clusters that corresponded to either up- or down regulated pathways (orange, green boxes). In **(B)** we calculated the change of expression correlation, ΔZ, for all pairs of OIMs in such clusters and their interacting leading edge genes in the corresponding pathways. Specifically, we observed multimodal distributions with local peaks around ΔZ = ±1.0 (dashed lines). In **(C)** and **(D)** we mapped all such interactions between OIMs and leading edge genes in these clusters if they had a correlation change |ΔZ|>1.0.
(PDF)

Table S1 **Utilized miR-mRNA interactions.** List of all 48,393 interactions between miRs and genes, that have been confirmed by two different sources (M: miRanda, P: PicTar, T: TargetScan).
(XLS)

Table S2 **Pairs of significantly associated miRs and pathways in glioblastomas.** 265 pathways that were significantly associated to miRs in glioblastoma. Each pathway is annotated with its leading edge genes and important miRs. P-Values are indicated in parentheses. Furthermore, we indicate if a pathway is differentially expressed (DE) with FDR<0.01.
(XLS)

Table S3 **Pairs of significantly associated miRs and pathways in ovarian cancer.** 322 pathways that were significantly associated to miRs in ovarian cancer. Each pathway is annotated with its leading edge genes and important miRs. P-Values are indicated in parentheses Furthermore, we indicate if a pathway is differentially expressed (DE) with FDR<0.01).
(XLS)

Table S4 **Pairs of significantly associated miRs and pathways in breast cancer.** 309 pathways that were significantly associated to miRs in breast cancer. Each pathway is annotated with its leading edge genes and important miRs. P-Values are indicated in parentheses. Furthermore, we indicate if a pathway is differentially expressed (DE) with FDR<0.01.
(XLS)

Table S5 **Pairs of miR/pathways that appear in all cancer types.** We show 98 pairs between 59 pathways and 27 miRs that appeared in GBMs, ovarian and breast cancers.
(XLS)

Table S6 **Significantly associated miRs in glioblastoma, ovarian and breast cancers.** In particular, we sorted miRs according to the number of significatly associated pathways (N_pw_) and annotaed each miR if they appear in a set of literature-curated cancer miRs (C), are differentially expressed (E) and implicated in the corresponding cancer type (I).
(XLS)
