# Supplementary figures and images for: Important miRs of Pathways in Different Tumor Types
Source: PLoS Comput Biol. 2013 Jan 24;9(1):e1002883. doi: 10.1371/journal.pcbi.1002883 (PMC3554575; doi:10.1371/journal.pcbi.1002883)

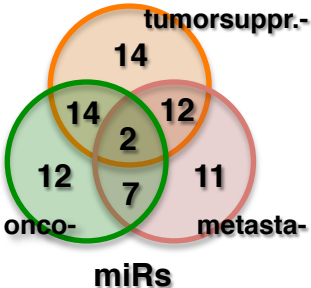

Supplement: Figure S1 — Overlaps of sets of onco-, tumorsuppressor- and metastamiRs. Venn diagram of the overlaps of 35 onco-, 42 tumorsuppressor- and 32 metastamiRs, totaling 72 cancer-related miRs. (PDF) [file pcbi.1002883.s001.pdf]

**A**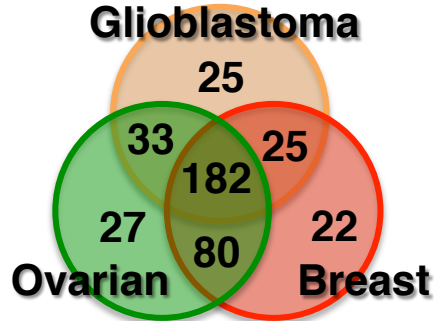**B**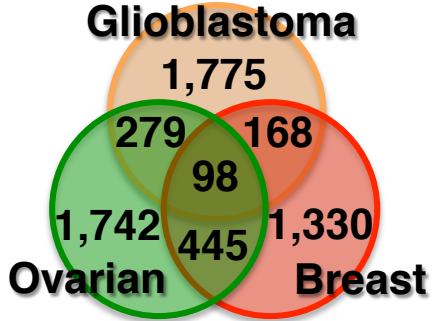

Supplement: Figure S2 — Overlaps of pathways and pairs of important miRs/pathways. (A) While we found 365 pathways in GBMs, 322 miRs in ovarian cancer and 309 in breast cancer (FDR<0.01), we observed large overlaps between these sets. (B) Focusing on overlapping pairs of important miRs and pathways that appeared in all cancer types considered, we observed a small overlap. (PDF) [file pcbi.1002883.s002.pdf]

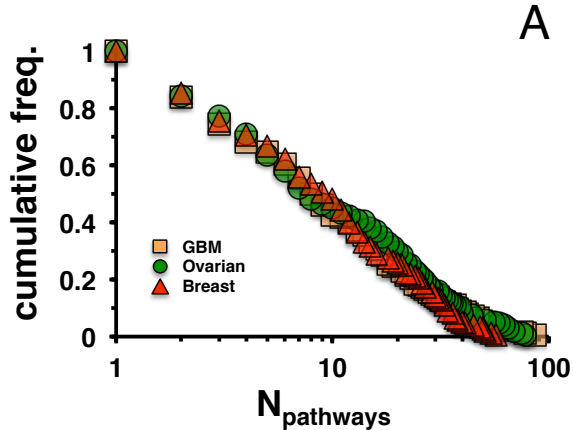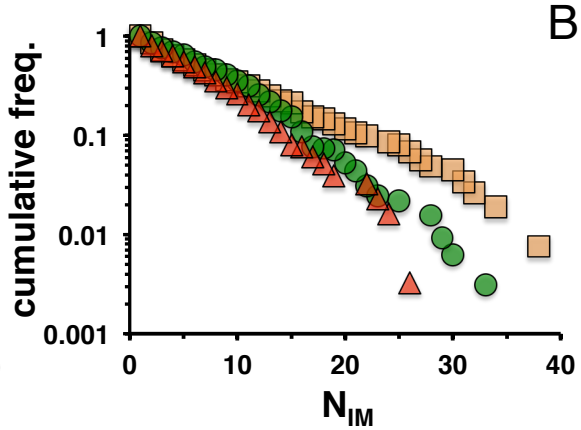

Supplement: Figure S3 — Statistics of important miRs and their pathways. In (A) we counted the number of pathways of each important miRs (IM), allowing us to find a logarithmic decay in such a frequency distribution. (B) In turn, we determined the number of IMs of each pathway, indicating an exponential decay in the corresponding frequency distribution. (PDF) [file pcbi.1002883.s003.pdf]

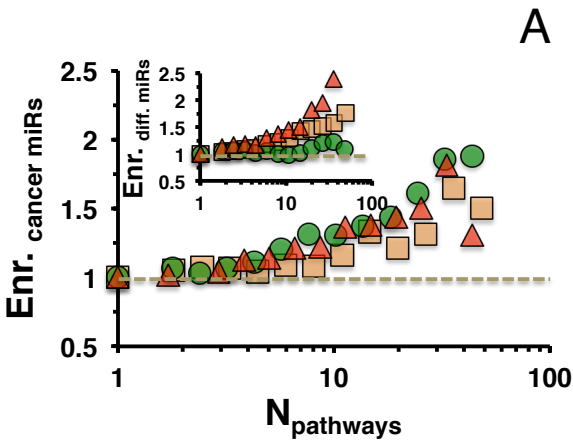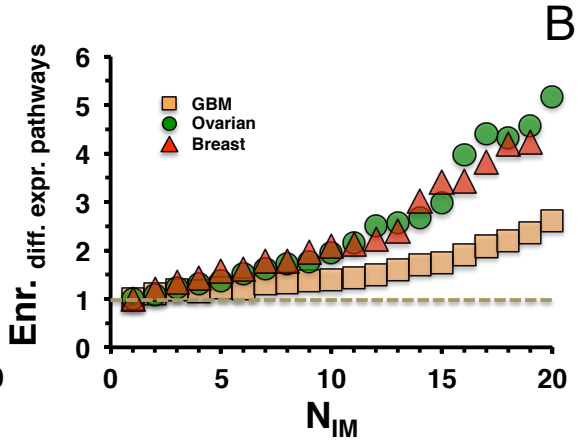

Supplement: Figure S4 — Enrichment analyses. In (A) we determined the enrichment of cancer miRs and differentially expressed miRs in groups of important miRs (IM) that have a certain number of pathways. Specifically, we observed that IMs with increasing number of pathways were enriched with literature curated cancer miRs as well as differentially expressed miRs (inset) in all cancer types considered. (B) In turn, we grouped pathways in sets that have at least a certain number of IMs. Determining the enrichment of differentially expressed pathways in such groups, we found that pathways with an increasing number of IMs tend to be differentially expressed. (PDF) [file pcbi.1002883.s004.pdf]

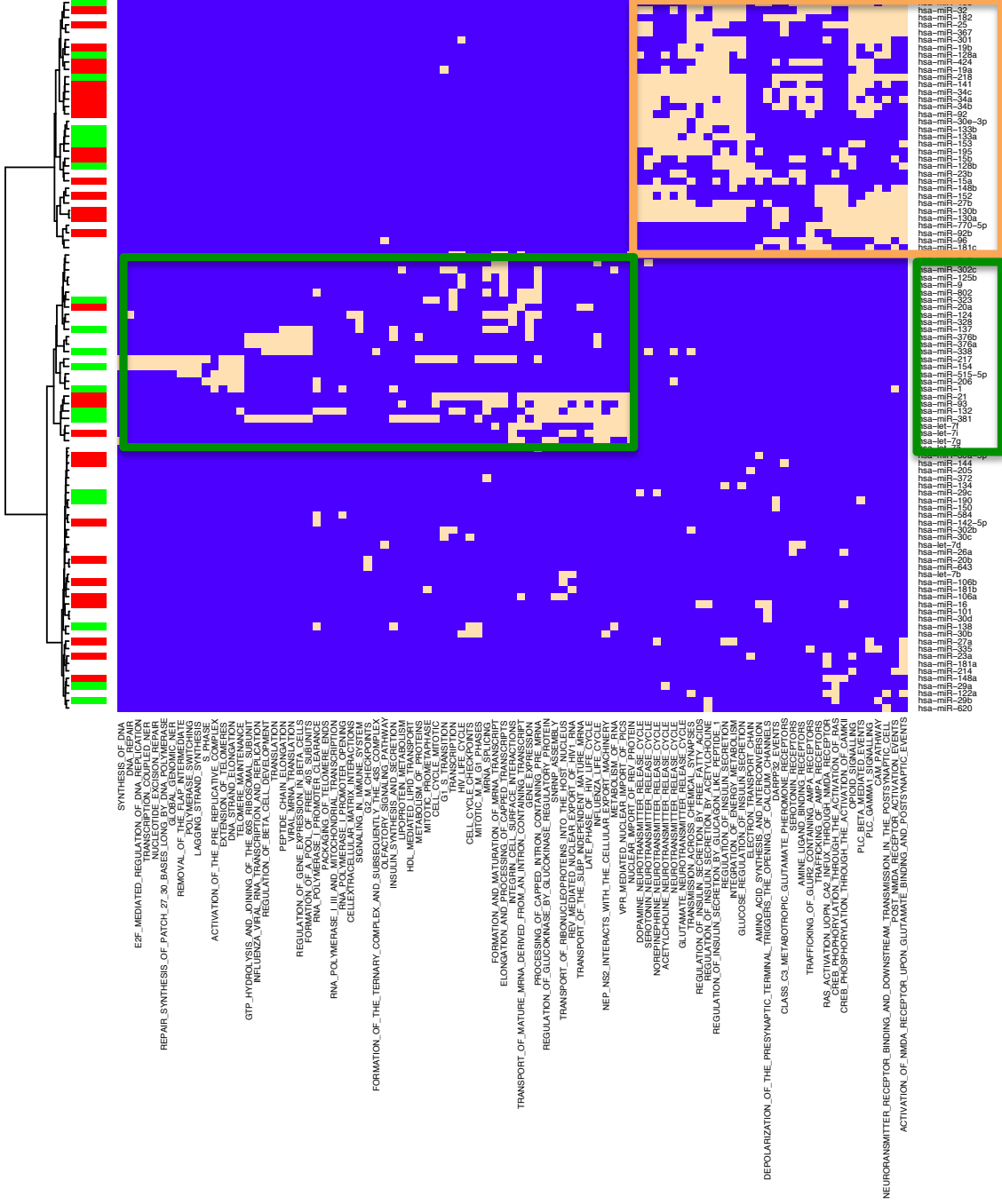

Supplement: Figure S5 — Enlargement of Fig. 3A in the main paper. (PDF) [file pcbi.1002883.s005.pdf]

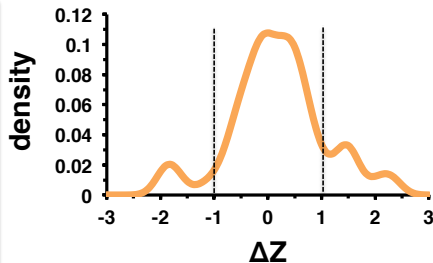

B

C

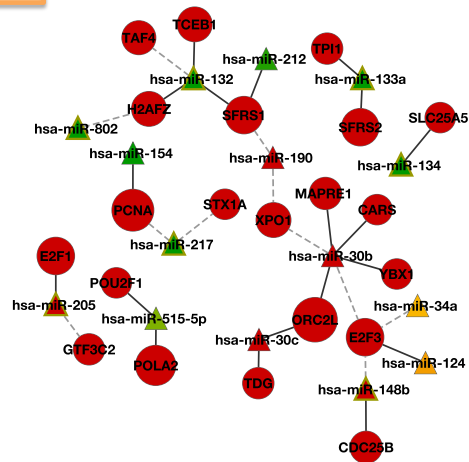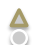

 (diff. exp.) miR  
 gene

+  
expr.

increasing number  
of pathways

— gain  $\Delta Z > 1$   
--- loss  $\Delta Z < -1$

Supplement: Figure S6 — Analysis of correlation change in ovarian cancer. (A) Focusing on our set of 99 overall important miRs (OIM), we indicated if such miRs were linked to differentially expressed pathways in ovarian cancer (peach boxes). We observed a large cluster that corresponded to down regulated pathways (orange box). In (B) we calculated the change of expression correlation, ΔZ, for all pairs of OIMs in this cluster and the interacting leading edge genes in the corresponding pathways, indicating local peaks around ΔZ = ±1.0 (dashed lines). In (C) we mapped all such interactions between OIMs and leading edge genes if they had a correlation change |ΔZ|>1.0. (PDF) [file pcbi.1002883.s006.pdf]

C

A

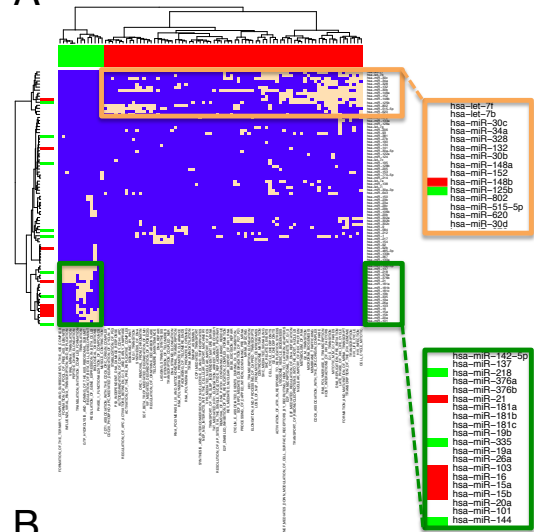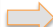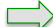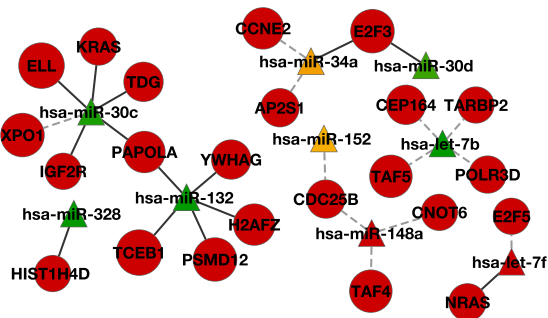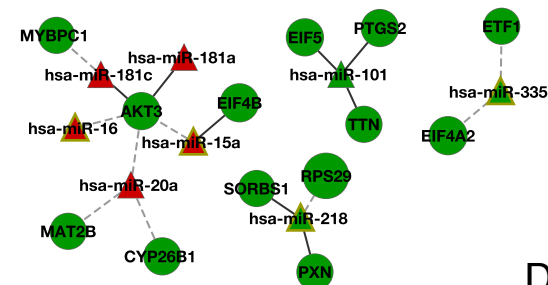

D

B

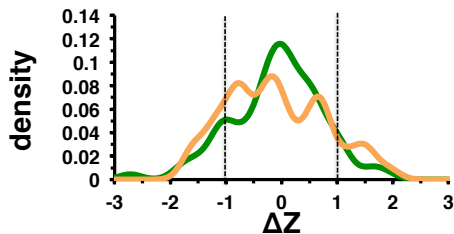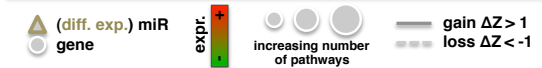

Supplement: Figure S7 — Analysis of correlation change in breast cancer. (A) Focusing on our set of 99 OIMs, we indicated if such miRs were linked to differentially expressed pathways in breast cancer (peach boxes). Specifically, we observed two large clusters that corresponded to either up- or down regulated pathways (orange, green boxes). In (B) we calculated the change of expression correlation, ΔZ, for all pairs of OIMs in such clusters and their interacting leading edge genes in the corresponding pathways. Specifically, we observed multimodal distributions with local peaks around ΔZ = ±1.0 (dashed lines). In (C) and (D) we mapped all such interactions between OIMs and leading edge genes in these clusters if they had a correlation change |ΔZ|>1.0. (PDF) [file pcbi.1002883.s007.pdf]
